# Mind-Body Medicine in Pediatrics

**DOI:** 10.3390/children4090076

**Published:** 2017-08-25

**Authors:** Hilary McClafferty

**Affiliations:** 1 Pediatric Integrative Medicine in Residency, University of Arizona Center for Integrative Medicine, Tucson, AZ 85724, USA; hmcclafferty@email.arizona.edu; 2 Department of Medicine, University of Arizona College of Medicine, Tucson, AZ 85724, USA

**Keywords:** mind-body medicine, new therapies, self-efficacy

## Abstract

The primary goals of this Special Issue are to encourage readers to become more familiar with the range of mind-body therapies and to explore their application in the pediatric clinical setting. The Special Issue includes a deliberate mix of case studies and practical clinical guidance, with the dual goals of piquing curiosity and providing resources for clinicians interested in pursuing further training.

The field of mind-body medicine holds significant promise for children and adolescents. Growing recognition of the intricate connections between thought, emotion, and physiology highlights the need for new therapies that leverage inner resources and offer non-invasive treatment options. Modern imaging techniques such as functional magnetic resonance imaging (fMRI) have advanced our understanding and appreciation of how the mind-body therapies can benefit health, increasing confidence in their use and propelling them into the mainstream of medicine. Mind-body therapies have special value in addressing pain, fear, and stress that accompanies many pediatric medical encounters. Some of the mind-body therapies such as mindfulness, have their roots in ancient traditions and are now being used routinely in the practice of pediatric integrative medicine, an emerging field focused on preventive health that blends conventional and evidence-based complementary therapies [1,2].

The primary goals of this Special Issue are to encourage readers to become more familiar with the range of mind-body therapies and to explore their application in the pediatric clinical setting. The Issue builds on the recent American Academy of Pediatrics Clinical Report: Mind-body Therapies in Children and Adolescents, which provides background and literature updates on several of the most commonly used mind-body therapies including biofeedback, clinical hypnosis, guided imagery, meditation, and yoga [3]. The Special Issue includes a deliberate mix of case studies and practical clinical guidance, with the dual goals of piquing curiosity and providing resources for clinicians interested in pursuing further training. 

A central theme of the Special Issue is the importance of enhancing self-efficacy in pediatric patients. The mind-body therapies are unique in that mastery can occur even in the very young, for example the use of relaxation exercises and self-hypnosis techniques in preschoolers. They are also highly versatile and are used in a range of clinical settings, from outpatient clinic to intensive care unit. A child who can acquire even an incremental sense of self-efficacy in a stressful environment will build resilience and ideally gain age-appropriate perspective that can build coping skills. The use of mind-body therapies may also facilitate emotional resilience in other venues, for example in school, sports, or performance arts. 

A corollary benefit of use of the mind-body therapies is the potential for stress reduction in the child’s caretakers, who can experience extraordinary stress while supporting a child through a medical experience. A third beneficiary are clinicians. The ability to provide options for treatment that are less invasive, cost-effective, and non-pharmacologic has potential to decrease clinician stress and hopefully contribute to reduction of burnout prevalence. 

The Issue’s contributing authors come from an impressive array of leading academic and private practice centers and are experts in their respective fields. They include specialists in research, infectious disease, psychology, adolescent medicine, neonatology, developmental-behavioral pediatrics, anesthesiology, pediatric intensive care, and general pediatrics. Topics covered touch on some of the most pressing concerns of clinicians and parents, including: childhood stress and trauma—especially related to adverse childhood events; acute, chronic, and perioperative pain; mental health; behavioral and developmental issues; and autoimmune illness such as inflammatory bowel disease.

The mind-body therapies discussed here can be used alone or blended with conventional therapies and include: mindfulness; medical yoga; technology assisted relaxation approaches such as biofeedback; cognitive-behavioral treatment; clinical hypnosis theory and application; neonatal massage; mind-body interventions in pediatric inflammatory bowel disease; mind-body therapies for children with attention deficit-hyperactivity disorder; and the emerging field of immersive virtual reality in pediatric pain patients. 

For some clinicians, considering the use of mind-body therapies stretches their definition of ‘real medicine’. This may be a result of training bias, personal experience, or insufficient familiarity with the accruing body of research in the field. I encourage readers to peruse this Special Issue with an open mind and, ideally, to entertain the question of not whether, but how the introduction of mind-body therapies into practice could benefit their patients and families. 
